# Spatio-temporal modelling of verotoxigenic *Escherichia coli* O157 in cattle in Sweden: exploring options for control

**DOI:** 10.1186/s13567-018-0574-2

**Published:** 2018-08-02

**Authors:** Stefan Widgren, Stefan Engblom, Ulf Emanuelson, Ann Lindberg

**Affiliations:** 1 Department of Disease Control and Epidemiology, National Veterinary Institute, 751 89 Uppsala, Sweden; 2 Division of Scientific Computing, Department of Information Technology, Uppsala University, 751 05 Uppsala, Sweden; 3 Department of Clinical Sciences, Swedish University of Agricultural Sciences, 750 07 Uppsala, Sweden

## Abstract

A spatial data-driven stochastic model was developed to explore the spread of verotoxigenic *Escherichia coli* O157 (VTEC O157) by livestock movements and local transmission among neighbouring holdings in the complete Swedish cattle population. Livestock data were incorporated to model the time-varying contact network between holdings and population demographics. Furthermore, meteorological data with the average temperature at the geographical location of each holding was used to incorporate season. The model was fitted against observed data and extensive numerical experiments were conducted to investigate the model’s response to control strategies aimed at reducing shedding and susceptibility, as well as interventions informed by network measures. The results showed that including local spread and season improved agreement with prevalence studies. Also, control strategies aimed at reducing the average shedding rate were more efficient in reducing the VTEC O157 prevalence than strategies based on network measures. The methodology presented in this study could provide a basis for developing disease surveillance on regional and national scales, where observed data are combined with readily available high-resolution data in simulations to get an overview of potential disease spread in unobserved regions.

## Introduction

Verotoxigenic *Escherichia coli* O157:H7 (VTEC O157) is a major causative agent of human enterohemorrhagic *E. coli* (EHEC) cases, with cattle being an important reservoir for the bacteria [1]. It has been suggested that a substantial proportion of the EHEC cases could be prevented if VTEC O157 shedding from cattle could be controlled [2, 3]. However, interventions aimed at reducing the VTEC O157 prevalence on a regional and national scale have so far not been successful. The distribution of VTEC O157 in the Swedish cattle population is not uniform in space and time; the prevalence is higher in southern Sweden [4, 5], and more cattle herds are infected during the autumn [6]. Risk-based surveillance is an approach to cost-efficiently identify infected premises by directing the sampling to those herds which are more likely to be infected [7]. Information regarding relevant risk factors for the pathogen on a geographical, herd, and individual level are required for planning such activities. A better understanding of the mechanisms behind the spatial and seasonal patterns of VTEC O157 infections in cattle could improve surveillance and prevention strategies.

Mathematical modelling and computer simulations provide means by which various assumptions and simplifications of pathogen spread in a complex system may be explored. A recent simulation study of VTEC O157 transmission involving the complete Swedish cattle population, showed that the spatial pattern in prevalence may be due to regional differences in livestock movements [8]. However, the model seemingly overestimated the prevalence of VTEC O157 in the northern regions. There are biologically plausible factors that could be incorporated in the model to improve the modelling accuracy and thus also our understanding. For example, it has been suggested that proximity to VTEC O157 infected farms is a risk factor for presence of VTEC O157 on farms because of local spread [6, 9, 10]. Hence heterogeneities in holding density might explain the different spatial pattern in prevalence. It has further been suggested that the seasonal variation in ambient temperature could have a considerable effect on the survival and growth of VTEC O157 in the environment and consequently on the indirect transmission dynamics [11, 12]. The cattle herd density is higher [13] and the duration of the warmer period longer [14] in the southern part of Sweden. These two parameters can be incorporated in the model using livestock and meteorological data.

It is commonly observed that VTEC O157 infection on farms cease without intervention [6, 15]. This implies that mitigation measures should rather strive at reducing the probability of introduction and decreasing the likelihood of spread, for example by increasing biosecurity, limit movement of infected cattle and reducing transmission from infectious animals to the environment by influencing shedding. Interventions aimed at reducing the impact of high-shedding individuals has been shown, in a modelling framework, to efficiently reduce the VTEC O157 prevalence [16]. However, to identify high-shedding individuals by faecal sampling is a challenge; the shedding pattern is highly variable, and it is possible that high-shedding events appear in many cattle for brief periods [17, 18]. Alternatively, mitigations could target farms that constitute a high risk for transmitting the infection. For example, using information from pig movement data to target control was reported to efficiently reduce the disease spread in simulations [19]. Simulations investigating interventions based on cattle movement data e.g. targeting highly connected herds, could further inform efficient control strategies for VTEC O157.

A mathematical model of infection dynamics in spatially segregated herds can be approached using a metapopulation model, i.e., a collection of subpopulations (herds) with their own infection dynamics [20]. To capture infection dynamics on a regional and national scale, the subpopulations must include interactions among them to model between-herd spread, for example, from livestock movements. Additionally, since each herd size is small, it is generally necessary to use stochastic models and run many iterations, for example, to account for the random event that an infection will go extinct [21]. Thus, performance of the simulator becomes critical when using these methods for national-scale disease spread modelling. There exist several recent approaches in computational epidemiology to implement such models, for example, KENDRICK [22], EMuLSion [23] and SimInf [24]. In the present study we used the SimInf framework which integrates infection dynamics in subpopulations as continuous-time Markov chains (CTMC) using the Gillespie stochastic simulation algorithm [25] and is designed to efficiently incorporate population demographics and livestock movement data.

The aims of this study were to use mathematical modelling and simulations to explore: (i) the importance of local spread of VTEC O157 among proximal holdings, (ii) the seasonal effect on the spatial distribution of VTEC O157 in Sweden, and (iii) the effectiveness of various interventions to reduce the prevalence of VTEC O157 in the Swedish cattle population.

## Materials and methods

### Input data

The complete Swedish livestock data for 37 221 holdings during the period 1 July 2005 to 31 December 2013 was incorporated in the simulations to drive the population demographics and the time-varying contact network from livestock movements. The data originated from the Swedish national cattle database managed by the Swedish Board of Agriculture. The dataset, described in detail in [8], contained 18 649 921 events reported on an individual animal level. Briefly, the livestock data included the following information about each cow: (i) the date and the holding for its birth, (ii) the date and the source and destination holding for any movements, and (iii) the date for slaughter or death. Moreover, all holdings (*n* = 37 221) were geopositioned using the available information with the highest spatial resolution for each holding. Exact coordinates were available for 31 188 holdings. For other farms, the 5-digit postal code was known for 4748 holdings and the locations were randomly sampled within the postal code area using the R package *sp* [26]. Similarly, for 1283 holdings the 3-digit postal area code was known (contains multiple postal codes) and each location was randomly sampled within the postal area. Finally, for two holdings the postal area code was missing. For these, other complementary information was used to determine the postal area to which they belonged. Random sampling of their coordinates was then performed in the postal areas. Each holding should correspond to a single geographical location, e.g. a farm building or a pasture, where animals are kept. However, some coordinates (*n* = 337) were identical between holdings. It was assumed that these coordinates correspond to e.g. a farm building and a nearby pasture managed by the same farmer. The distance *d*_*ik*_ was calculated for every holding *i* to all neighbours *k*. A minimum distance of 100 m was used to separate any two holdings to avoid division by zero in Equation (2) below.

Data from the Swedish Meteorological and Hydrological Institute (SMHI) was used to determine the duration of the seasons (winter, spring, summer, and autumn) depending on geographical location. The seasons were defined by SMHI per the average temperature for the reference period 1961–1990: (winter) below 0 °C, (spring) between 0 and 10 °C, (summer) above 10 °C and (autumn) between 0 and 10 °C [14]. Determined by the location, each holding had its own definition of when (day of the year) each season started. Note that this approach does not incorporate a year-to-year variation in when the seasons begin.

The dataset that was used to calibrate the model parameters (described below), was from a longitudinal herd-level observational study conducted over 38 months (2009–2012) in 126 cattle herds in four distinct regions in southern Sweden [6]. In that study, the VTEC O157 herd status was repeatedly determined, on average at 17 occasions 64 days apart. VTEC O157 was detected in 67 of the herds on 224 occasions. The VTEC O157 herd status was determined from environmental sampling among calves (younger than 120 days) and young stock (between 120 and 365 days).

To evaluate the simulated spatial distribution of infected holdings, it was visually compared with the observed distribution of a nation-wide monitoring of VTEC O157 in Swedish cattle that was carried out in abattoirs from autumn 2011 to autumn 2012 [27]. In total, the pathogen was detected in 73 out of 2376 randomly collected faecal samples. Moreover, the farm that each sampled animal originated from was geopositioned (Figure 3).

### Pathogen transmission model

The VTEC O157 transmission was modelled using an extended variant of the stochastic compartment model SIS_E_ described elsewhere [8, 28]. The model contained the compartments susceptible (S) and infected (I) as well as the environmental compartment (E), contaminated with free living pathogens by infected and shedding animals. The susceptible and infected compartments were further divided into three age categories to match the age categories in the longitudinal herd-level observational study [6] used for calibration. Let index *j* denote the age category where: (1) calves—younger than 120 days, (2) young stock—between 120 and 365 days, and (3) adults—older than 365 days.

The transitions between the susceptible and infected compartments were modelled as a continuous-time discrete state Markov chain (CTMC). The transition from susceptible to infected depends on the concentration of the environmental infectious pressure ${\varphi_i}(t)$ in holding *i* and the age dependent indirect transmission rate υ_*j*_. The environmental infectious pressure ${\varphi_i}(t)$ was assumed to be uniformly distributed within each holding and depend on the amount of bacteria excreted by infected individuals. Since the floor surface area is unknown, for simplicity, it was assumed to be proportional to the number of individuals in each holding. The transition from infected to susceptible depends on the recovery rate *γ*.

1 $$\begin{aligned} S_{ij} \mathop{\longrightarrow}\limits^{{\upsilon_{j} \varphi_{i} }}I_{ij} \\ I_{ij} \mathop{\longrightarrow}\limits^{\gamma }S_{ij} \\ \end{aligned}$$

The model was extended from the previous version [8, 28] to include a spatial coupling among proximal holdings to capture other transmission routes unrelated to moving infected animals. It was assumed that indirect transmission relative to the environmental infectious pressure ${\varphi_i}(t)$ occurs among proximal holdings, for example, by birds and rodents, and that the magnitude of this exchange decreased with the Euclidean distance within a radius r = 5000 m. The distance 5000 m was used, since having infected neighbouring farms within that distance was associated with an increased risk for VTEC infection in the longitudinal herd-level observational study [6] used for calibration. Let *D* denote the rate of the coupling, and let *d*_*ik*_ denote the distance between the two holdings *i* and *k*. Furthermore, let *S*_*i*_ and *I*_*i*_ denote the number of susceptible and infected in holding *i*, respectively, and *N*_*i*_ = *S*_*i*_ + *I*_*i*_ the herd size. The time dependent environmental infectious pressure ${\varphi_i}(t)$ was modelled with an ordinary differential equation as

2 $$\frac{{d\varphi_{i} \left( t \right)}}{dt} = \frac{{\alpha I_{i} \left( t \right)}}{{N_{i} \left( t \right)}} + D\mathop \sum \limits_{k} \frac{{\varphi_{k} \left( t \right)N_{k} \left( t \right) - \varphi_{i} \left( t \right)N_{i} \left( t \right)}}{{d_{ik} N_{i} \left( t \right)}} - \beta \left( t \right)\varphi_{i} \left( t \right)$$

for all *i* ≠ *k* such that *d*_*ik*_ ≤ *r*. Furthermore, *α* denotes the average daily rate of contribution to the environmental infectious pressure, per infected individual. Finally, *β*(*t*) denotes the rate of the decay of the environmental infectious pressure in a holding.

To incorporate seasonality in the infection dynamics, *β* could vary during the year. However, compared to the model in [8], where the year was evenly divided into four quarters, it was instead divided into the four seasons: spring, summer, autumn and winter, with *β*_1_, *β*_2_, *β*_3_, and *β*_4_ reflecting the rate of decay in each season, respectively. In this way, the parameter *β* could indirectly capture the effect of varying seasons.

### Model calibration

The disease spread model was calibrated against observed longitudinal data from 126 holdings [6]. The vector of model parameters (Table 1), defined as $\varvec{\theta}$, was adjusted to find the set of parameter values that minimised the difference between the observed and the simulated outcome for the 126 holdings. The agreement was measured using the *objective function*

3 $$G\left(\varvec{\theta}\right) = G_{1} \left(\varvec{\theta}\right) + G_{2} \left(\varvec{\theta}\right)$$

with quantity $G_{1} \left(\varvec{\theta}\right)$ as a MSE (mean squared error) associated to the number of infected holdings aggregated quarterly, and the quantity $G_{2} \left(\varvec{\theta}\right)$ as a MSE associated to the number of newly infected holdings quarterly. This metric was chosen to recognise that disease dynamics in prevalence involves recovery and incidence.

**Table 1:** **Parameters in a stochastic SIS**_**E**_ **VTEC O157 model**

Parameter	Description (unit)	Value
Α	Rate of contribution to the environmental infectious pressure per infected individual (units per day)	^a^1.00 × 10^0^
*β*_1_	Decay of environmental infectious pressure during spring (per day)	1.57 × 10^−1^
*β*_2_	Decay of environmental infectious pressure during summer (per day)	^a^1.44 × 10^−1^
*β*_3_	Decay of environmental infectious pressure during autumn (per day)	1.50 × 10^−1^
*β*_4_	Decay of environmental infectious pressure during winter (per day)	1.57 × 10^−1^
*υ*_1_	Indirect transmission rate of the environmental infectious pressure in calves (per animal per day)	2.48 × 10^−2^
*υ*_2_	Indirect transmission rate of the environmental infectious pressure in young stock (per animal per day)	2.48 × 10^−2^
*υ*_3_	Indirect transmission rate of the environmental infectious pressure in adults (per animal per day)	1.37 × 10^−2^
*γ*	The recovery rate of infection (per day)	^a^1.00 × 10^−1^
D	The rate of local spread among proximal holdings (per day per m)	0.11 × 10^−5^

Parameters used to explore the spread of verotoxigenic *Escherichia coli* O157:H7 (VTEC O157) in the entire Swedish cattle population based on data reported to the Swedish Board of Agriculture during the period 01 July 2005 to 31 December 2013. The within-herd disease spread was modelled with a stochastic SIS_E_ compartment model with the two disease states: susceptible (S) and infected (I) and E representing the environmental compartment contaminated with VTEC O157 by infected animals. The decay of the environmental infectious pressure was varied in each of the four seasons: spring, summer, autumn, and winter. Individuals were divided into the following three age categories; *calves* 0–119 days, *young stock* 120–364 days and *adults* older than 364 days.

^a^Fixed value during model fit.

The longitudinal herd-level observational study [6] was repeatedly replicated in simulations to determine $G_{1} \left(\varvec{\theta}\right)$ and $G_{2} \left(\varvec{\theta}\right)$, as follows. Let $Y_{in}^{*}$ denote the *n*th observed VTEC O157 status (1-positive; 0-negative) in holding *i* at time *t*_*n*_. Similarly, let $Y_{in} \left(\varvec{\theta}\right)$ denote the simulated status, corresponding to $Y_{in}^{*}$. To determine the status $Y_{in} \left(\varvec{\theta}\right)$ that could have been found if simulated holdings had been sampled, the sampling strategy from the observed data was replicated as previously described [8]. Briefly, the sampling was simulated at each sample point as follows. First, pools (pool size = 3) were randomly created within each age category from the number of susceptible and infected individuals at the time for the sample point in the simulation. Given the proportion of infected individuals in a pool, it was randomly classified as positive or negative, with P (positive) equal to the estimated test sensitivity [29]. Finally, using the estimated pool prevalence, the simulated herd status was randomly classified as positive or negative given the sensitivity of the sampling protocol [30].

To evaluate $G_{1} \left(\varvec{\theta}\right)$, the statuses $Y_{in}^{*}$ and $Y_{in} \left(\varvec{\theta}\right)$ were aggregated quarterly from Q4 in 2009 to Q4 in 2012, yielding 13 groups $Q = \left\{ {Q4_{2009} ,Q1_{2010} , \ldots , Q4_{2012} } \right\}$ (Q1-January–March; Q2-April–June; Q3-July–September; Q4-October–December). $G_{1} \left(\varvec{\theta}\right)$ was then defined to quantify the differences in the number of observed and simulated infected holdings per quarter, as follows. Let $a_{q}^{*}$ and *a*_*q*_ denote the number of observed and simulated statuses $Y_{in}^{*}$ and $Y_{in} \left(\varvec{\theta}\right)$ in quarter *q*, respectively,

4 $$a_{q}^{*} = \mathop \sum \limits_{{t_{in} \in q}} Y_{in}^{*} ,$$

5 $$a_{q} = \mathop \sum \limits_{{t_{in} \in q}} Y_{in} \left(\varvec{\theta}\right),$$

where *q* ∈ *Q*. The objective function $G_{1} \left(\varvec{\theta}\right)$ was then defined as

6 $$G_{1} \left(\varvec{\theta}\right) = \frac{{\mathop \sum \nolimits_{q \in Q} \left( {\overline{a}_{q} - a_{q}^{*} } \right)^{2} }}{{\sigma_{{a_{q}^{*} }}^{2} }}$$

where the MSE was scaled with the variance $\sigma_{{a_{q}^{*} }}^{2}$ of the observed data and the coefficients *a*_*q*_ were averaged over *N* = 40 trajectories to account for that each outcome from the stochastic simulator provided a different measurement of the system,

7 $$\overline{a}_{q} = \frac{1}{N}\mathop \sum \limits_{j = 1}^{N} a_{q,j} .$$

To measure $G_{2} \left(\varvec{\theta}\right)$, the incident cases were counted, per quarter, i.e. the number of new holdings with a positive status each quarter of the year, as follows. Let $X_{in}^{*}$ denote the first occurrence of a positive status in holding *i* at time *t*_*n*_, such that $X_{in}^{*} = 1$ at the first occasion where $Y_{in}^{*} = 1$, and $X_{in}^{*} = 0$ for all other occasions in the time series for holding *i*. Similarly, let $X_{in} \left(\varvec{\theta}\right)$ denote the simulated incident cases, corresponding to $X_{in}^{*}$. Let $b_{q}^{*}$ and *b*_*q*_ denote the number of observed and simulated incident cases $X_{in}^{*}$ and $X_{in} \left(\varvec{\theta}\right)$ in quarter *q*, respectively,

8 $$b_{q}^{*} = \mathop \sum \limits_{{t_{in} \in q}} X_{in}^{*} ,$$

9 $$b_{q} = \mathop \sum \limits_{{t_{in} \in q}} X_{in} \left(\varvec{\theta}\right),$$

where *q* ∈ *Q*. The objective function $G_{2} \left(\varvec{\theta}\right)$ was defined as

10 $$G_{2} \left(\varvec{\theta}\right) = \frac{{\mathop \sum \nolimits_{q \in Q} \left( {\overline{b}_{q} - b_{q}^{*} } \right)^{2} }}{{\sigma_{{b_{q}^{*} }}^{2} }},$$

where, similarly the MSE was scaled with the variance $\sigma_{{b_{q}^{*} }}^{2}$ of the observed data and the counts *b*_*q*_ were averaged over *N* = 40 trajectories

11 $$\overline{b}_{q} = \frac{1}{N}\mathop \sum \limits_{j = 1}^{N} b_{q,j} .$$

The Nelder–Mead algorithm [31] in R [32] was used to find the parameter combination $\varvec{\theta}$ that minimised the objective function $G\left(\varvec{\theta}\right)$ under the constraint that all parameters $\varvec{\theta}\ge 0$. Exploratory analysis indicated that fitting a model with the parameters *υ*_1_, *υ*_2_, *υ*_3_, *β*_1_, *β*_2_, *β*_3_, *β*_4_, and *D*, was not feasible i.e. the system was not identifiable. Therefore, to maintain model parsimony, *υ*_1_ and *υ*_2_ (calves and young stock indirect transmission rates) were fitted as a single parameter, and *β*_2_ (summer decay) was fixed at a decimal reduction rate (the time required at a given temperature to kill 90% of VTEC O157) of 16 days [33–35]. Moreover, the average recovery rate was fixed at γ = 0.1 [36]. Finally, the contamination rate *α*, was fixed at 1.0 per day, thus defining the unit of the environmental infectious pressure variable ${\varphi_i}(t)$. The disease spread simulations were performed with the SimInf package in R [8, 21, 24, 37].

The Nelder–Mead algorithm was started from two different parameter conditions and then restarted a third time from the parameters found for the minimum $G\left(\varvec{\theta}\right)$. Each Nelder–Mead optimisation ran for 250 iterations. To quantify stochasticity in $G\left(\varvec{\theta}\right)$, the coefficient of variation was calculated from the last 10 iterations of the Nelder–Mead.

### Initialisation

Simulations started 1 July 2005 with a uniform geographical distribution with 25% of the holdings randomly sampled to be infected. To have an overall individual prevalence of 4% [5], the within-holding prevalence in initially infected holdings was set to 16% in all age categories. Moreover, the initial environmental infectious pressure ${\varphi_i}$ was set to zero in each holding. The model was simulated for a burn-in of 1570 days before the parameter estimation started in October 2009.

### Evaluation of the calibrated model

Using the calibrated model parameters, the model was graphically evaluated based on the outcome of 1000 trajectories. First, the distributions of the simulated data $\overline{a}_{q}$ and $\overline{b}_{q}$ in each quarter were plotted in a boxplot and compared with the observed data $a_{q}^{*}$ and $b_{q}^{*}$. Secondly, distributions of weekly time-series of the herd-level prevalence and the individual-level prevalences in each age category were generated for the period 1 January 2008 to 31 December 2013. Finally, the spatial distribution of the proportion of trajectories where each holding had at least one infected animal was visualised for the following dates: 1 October 2011, 1 January 2012, 1 April 2012, and 1 July 2012. The spatial distribution was compared with the outcome from the nation-wide monitoring of VTEC O157 in Swedish cattle 2011–2012 [27].

### Sensitivity analysis

Sensitivity analysis was performed to explore how perturbations of the six calibrated model parameters (*υ*_1,2_, *υ*_3_, *β*_1_, *β*_3_, *β*_4_, *D*) would influence $G\left(\varvec{\theta}\right)$. First, each parameter was perturbed at multiple values, keeping the others fixed at the calibrated value. For every perturbation, the average $G\left(\varvec{\theta}\right)$ was estimated from 40 trajectories. Secondly, a global sensitivity analysis was conducted since there may be interactions among parameters, thus affecting $G\left(\varvec{\theta}\right)$. The Extended Fourier Amplitude Sampling Test (eFAST) [38, 39] was used for the global sensitivity analysis. This measure gives an indication of the influence of each parameter and groups of parameters on $G\left(\varvec{\theta}\right)$. Based on the exploratory perturbation analysis above, the parameters were constrained to the following respective range: *υ*_1,2_ (0.022–0.027), *υ*_3_ (0.012–0.015), *β*_1,3,4_ (0.13–0.2), and *D* (0–0.3). Additionally, a “dummy” (1–10) parameter that does not affect the simulation was added for statistical comparison. The eFAST analysis was performed using 65 samples per search curve and a resampling size of 3 and 40 replicates per parameter set. The spartan R package [40], was used to generate the 1365 parameter value sets and for analysing the response in $G\left(\varvec{\theta}\right)$. The first-order (Si) and total-order (STi) sensitivity indexes were calculated for each parameter and plotted. A two-sample t-test (significance level 0.95) was estimated to indicate significance of each parameters sensitivity index, contrasted to the “dummy” parameter.

### Implementation of control strategies

The effectiveness of control strategies was investigated from numerical experiments comparing a baseline, i.e. the outcome from simulations with the calibrated model parameters, with the outcome from simulations with an adjusted model that reflected the control scenario in question. One thousand trajectories were generated for each investigated control scenario, and weekly time series of the average herd-level prevalence were generated for the period 1 January 2008 to 31 December 2013. All control scenarios started 1 January 2009.

First, control scenarios aimed at reducing the between-herd transmission were investigated. One approach to limit connectivity between herds is simply to remove some livestock movements from the data. However, removing the movements will affect the demography. Therefore, infected animals about to be moved, were replaced with susceptible animals, leading to the same results for the risk of infection, but keeping the demography in adequacy with the observed data. Two network-based strategies were explored in the simulations to inform when to apply control. One strategy was to reduce the number of holdings from which an infection could be introduced. This was done by considering a threshold for applying control based on the in-degree (ID) [41] and the ingoing contact chain (ICC) [42] of the receiving holding. The ID of a holding is defined as the number of holdings that move animals directly to the holding for a defined period. The ICC metric additionally includes all holdings that have indirect movements i.e. all holdings that can be reached when tracing the sequence of movements over time. Similarly, the other strategy was to reduce the number of holdings to which an infection could spread. This was done by instead applying control based on the out-degree (OD) [41] and the outgoing contact chain (OCC) [43] of the sending holding. The OD of a holding is defined as the number of holdings that the holding moves animals to for a defined period. The OCC metric additionally includes all holdings that can be reach indirectly when tracing the sequence of movements over time. The ID, OD, ICC and OCC over 90 days were calculated weekly for each holding during the period 1 July 2005 to 31 December 2013 using the R package EpiContactTrace [44]. After inspecting the 80–97.5% percentiles of ID, OD, ICC and OCC summarised for the whole period and over all holdings, the following thresholds were considered for ID and OD: >1, >2, >3 and >4, and for ICC and OCC: >2, >4, >6, >8 and >10. In the simulations, the model simply changed the state of an infected individual to susceptible when it was moved, conditional on the weekly network measures, so that infection was not transferred to the destination holding [37].

Next, control scenarios aimed at reducing the animal-to-animal transmission within a herd were explored. A reduction of the between-animal transmission can be accomplished by making cattle less likely to become infected and excrete the pathogen. One approach to achieve this is to use vaccination [reviewed by 45, 46], which has been shown to inhibit VTEC O157 colonisation of the terminal recta and thereby leading to a significant reduction in shedding. Another approach is to adjust the microflora of the gastrointestinal tract to prevent colonisation of VTEC O157 by feeding probiotics [reviewed by 47]. Although the complex mechanisms involved in treatments, such as vaccination or probiotics, are not specifically included in the model, it was assumed that modifying the parameters α (rate of contamination to the environment) and *υ* (indirect transmission rate) would mimic the effect of such a treatment. The following parameter adjustments were considered to explore effects of reduced animal-to-animal transmission: (i) 10% reduction of the contamination rate *α*, (ii) 10% reduction of the indirect transmission rate *υ*, and (iii) both (i) and (ii) together.

Finally, although unrealistic to consider for control, scenarios were also generated to compare the outcome when blocking all between-holding transmission routes via: (i) livestock movements, (ii) the spatial coupling among proximal holdings, and (iii) both (i) and (ii).

## Results

### Model calibration

The model reached a minimum at $G\left(\varvec{\theta}\right) = 16.75$, where $G_{1} \left(\varvec{\theta}\right) = 6.42$ and $G_{2} \left(\varvec{\theta}\right) = 10.33$ for the parameter values of $\varvec{\theta}$ shown in Table 1. The coefficient of variation from 10 replicates of $G\left(\varvec{\theta}\right)$ was estimated to 2.85%. The indirect transmission rate was higher for animals under 1 year of age compared to older animals in the calibrated model. Furthermore, the highest rate of the bacterial reduction per day was during the winter and spring seasons and the least reduction was during the summer.

**Figure 1:**
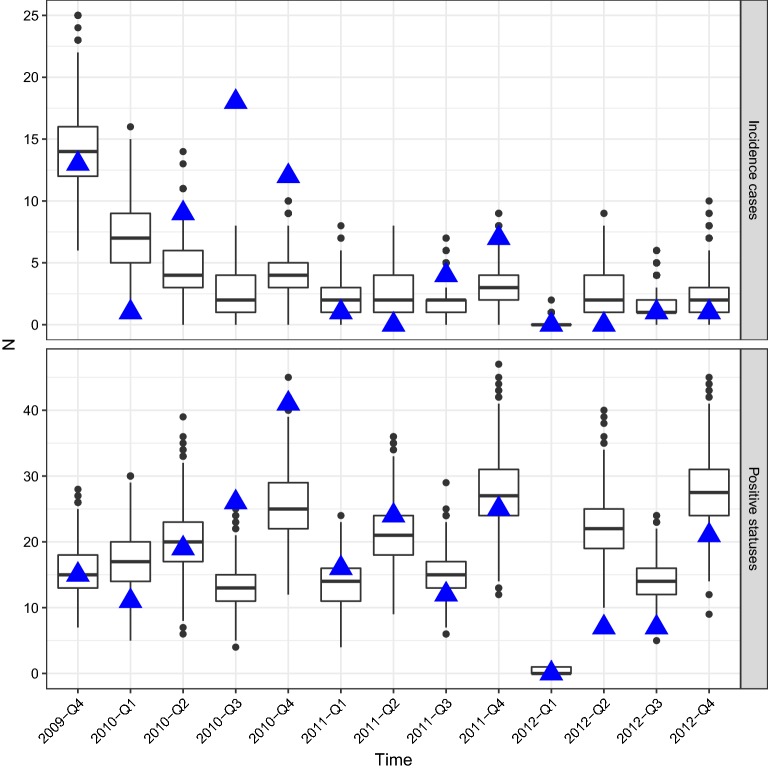
**Evaluation of model fit.** Comparison between the observed verotoxigenic *Escherichia coli* O157:H7 herd status in a longitudinal observational study from October 2009 to December 2012 (38 months) in 126 cattle herds [6] (blue filled triangle) and simulated data that replicated that study in 1000 trajectories (boxplot). The figure shows the number of incident cases by quarter i.e. new herds that were found positive in each quarter, and the number of positive herds in each quarter, in total. Q1: January–March, Q2: April–June, Q3: July–September, and Q4: October–December.

Figure 1 compares the number of positive herds and incident cases each quarter in the observed data [6] and simulated data, based on 1000 trajectories. The observed count was within the range of simulated count in most quarters (22 of 26 observations) and within the interquartile range in 10 of 26 observations. The simulated range for incident cases underestimated the observed counts in Q3 and Q4 2010 and overestimated the observed counts in Q1 2010. Moreover, the simulated data overestimated the number of positive herds in Q2 2012 (Figure 1). As can be seen in Figure 1, the simulated data does not capture the variation of the observed data.

**Figure 2:**
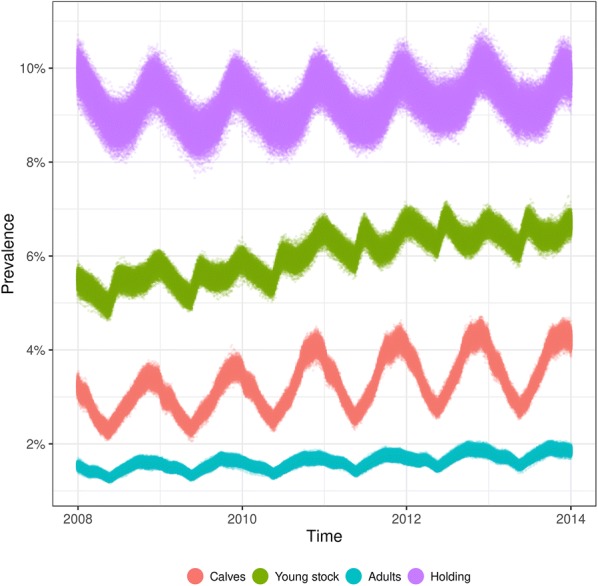
**Distribution of holding-level and individual-level prevalences.** Stochastic disease spread modelling of verotoxigenic *Escherichia coli* O157:H7 in the complete Swedish cattle population. Calves are younger than 120 days, young stock are between 120 and 365 days, and adults are older than 365 days. The holding prevalence was calculated among the number of active holdings i.e. holdings having at least one animal. The prevalences were determined from 1000 trajectories.

The calibrated model had seasonal prevalence patterns at the holding- (mean: 9.3%, range 7.7–10.9%) and individual level that peaked around the end of each year (Figure 2). The individual level prevalence was highest in young stock (mean: 6.0%, range 4.6–7.3%), then in calves (mean: 3.3%, range 2.0–4.7%), and lowest in adults (mean: 1.6%, range 1.2–2.1%).

**Figure 3:**
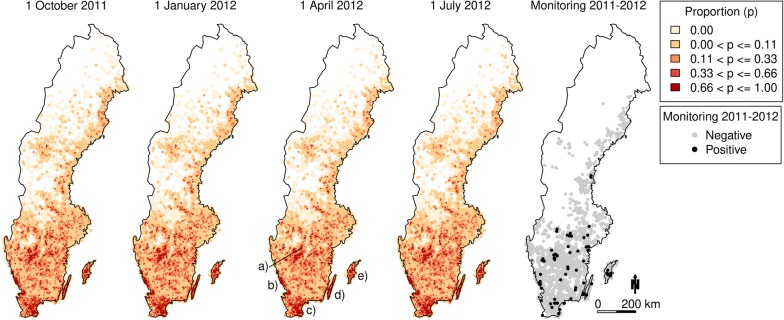
**Distribution of infected holdings.** (Left) Stochastic disease spread modelling of verotoxigenic *Escherichia coli* O157:H7 (VTEC O157) in the complete Swedish cattle population. The four left figures show the proportion of 1000 simulated trajectories that each holding had at least one infected animal at 1 October 2011, 1 January 2012, 1 April 2012, and 1 July 2012. The right figure shows a comparison of individual cattle VTEC O157 status from samples collected at abattoirs 2011–2012, where the circles represent the origin of each sampled animal. To reduce over-plotting, jitter was added to the coordinates. Letters denote regions: (a) Falköping, (b) Halland, (c) Skåne, (d) Öland and (e) Gotland.

Figure 3 shows the spatial distribution of the proportion of 1000 trajectories in which each holding had one or more infected animals at 1 October 2011, 1 January 2012, 1 April 2012, and 1 July 2012. The main clusters of infected holdings were in the south (Skåne), south-west (Halland), the two south-east islands (Öland, Gotland), and in the western inland (Falköping), see Figure 3. The appearance is essentially identical for each of the four dates. The spatial distribution of the main clusters in the simulated data is in agreement with the results from the nation-wide monitoring of VTEC O157 in Swedish cattle 2011–2012 [20].

### Sensitivity analysis

**Figure 4:**
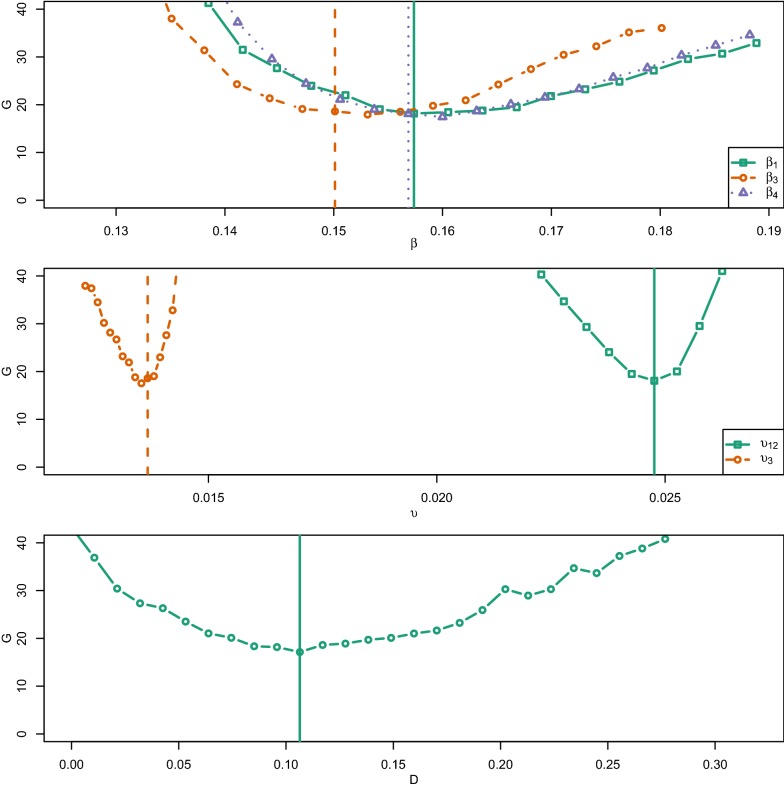
**One at a time variation sensitivity analysis of the objective function G**(**θ**). Exploratory analysis of the objective function G(θ) that measured the agreement between the observed verotoxigenic *Escherichia coli* O157:H7 herd status in a longitudinal observational study from October 2009 to December 2012 (38 months) in 126 cattle herds and a stochastic disease spread model that replicated that study. The graphs show the response in objective function $G\left({\theta}\right)$ when varying one parameter (*υ*_1,2_, *υ*_3_, *β*_1_, *β*_3_, *β*_4_, and *D*) at a time, keeping the other parameters fixed at the calibrated value (Table 1). For each parameter value (indicated with an open circle, square or triangle), the average G(θ) was calculated from 40 trajectories. Vertical lines indicate respective calibrated parameter value.

Figure 4 shows the result from the sensitivity analysis of the objective function $G\left(\varvec{\theta}\right)$ when varying one parameter (*υ*_1,2_, *υ*_3_, *β*_1_, *β*_3_, *β*_4_, and *D*) at a time, keeping the other parameters fixed at the calibrated value. For each parameter, $G\left(\varvec{\theta}\right)$ increased when the parameter was either increased or decreased. The response was most distinct for small changes in the parameters values of *υ*_1,2_ and *υ*_3_. In contrast, a ± 100% change of the spatial coupling parameter D marginally increased $G\left(\varvec{\theta}\right).$

**Figure 5:**
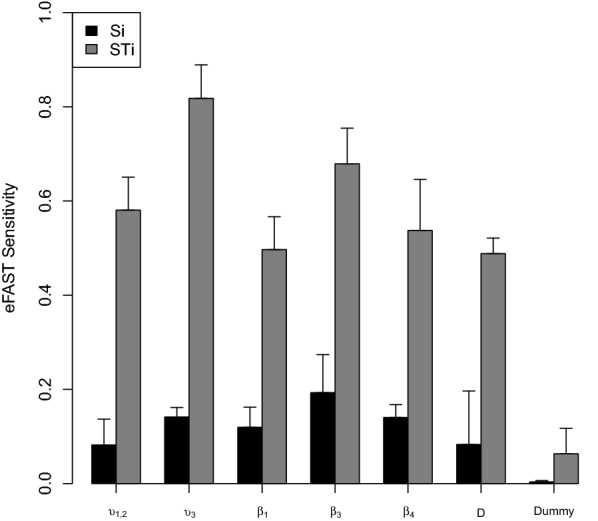
**Global sensitivity analysis of the objective function G**(**θ**). Partitioning of variance using Extended Fourier Amplitude Sampling Test (eFAST) on the objective function G(θ) that measured the agreement between the observed verotoxigenic *Escherichia coli* O157:H7 herd status in a longitudinal observational study from October 2009 to December 2012 (38 months) in 126 cattle herds and a stochastic disease spread model that replicated that study. The first-order (Si) sensitivity indexes (black bars) describes variance explained by the value assigned to that parameter. The total-order (STi) sensitivity indexes (grey bars) describes variance caused by this parameter and non-linear effects between this parameter and others. Error bars are standard error over three resample curves.

Figure 5 shows the result from the eFAST analysis. The first-order (Si) sensitivity indexes were statistically significant (*p* < 0.05) for the parameters: indirect transmission rate in adult cattle (*υ*_3_), and the decay of the environmental infectious pressure in the spring (*β*_1_) and winter (*β*_4_). The total-order (STi) sensitivity indexes were statistically significant (*p* < 0.05) for all calibrated parameters (*υ*_1,2_, *υ*_3_, *β*_1_, *β*_3_, *β*_4_, and *D*). All STi indexes dominated over Si indexes.

### Implementation of control strategies

**Figure 6:**
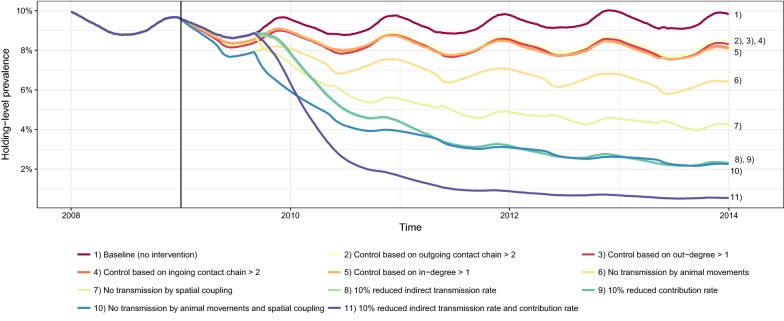
**Effect on holding-level prevalence.** Comparison between various simulated intervention strategies, starting at 1 January 2009 (vertical line), to reduce the holding-level prevalences (averaged over 1000 trajectories) of verotoxigenic *Escherichia coli* O157:H7 in the complete Swedish cattle population. The numbered items in the legend are indicated in the plot, where the items are ordered from highest to lowest prevalence at 31 December 2013. Please note that lines for some prevalences have a high degree of overlap.

Figure 6 shows the holding-level prevalence after the different control strategies had been applied since 1 January 2009 (5 years) to reduce spread of VTEC O157. Control based on the network measures ID (cut-point > 1), OD (cut-point > 1), ICC (cut-point > 2) and OCC (cut-point > 2) marginally reduced the prevalence. The reduction achieved with the other investigated cut-points of ID, OD, ICC and OCC was even less (data not shown). Reducing the average rate of contribution to the environmental infectious pressure (*α*), or the indirect transmission rate (*υ*_1,2_ and *υ*_3_) with 10%, efficiently decreased the prevalence. The decrease was further pronounced when reducing *α*, *υ*_1,2_ and *υ*_3_ in combination. For the extreme scenarios, where animal movements and/or spatial coupling was completely blocked, the holding-level prevalence clearly decreased, however, not to same extent as when *α*, *υ*_1,2_ and *υ*_3_ were reduced in combination.

## Discussion

The present simulation study assumed that between-herd disease transmission of VTEC O157 occurs by moving infected animals and that there also exists spatial spread among proximal herds. In addition, it was assumed that season affects the survival of VTEC O157 and thus infection dynamics. The results show that the location of geographical regions with the most pronounced clustering of infected holdings was in agreement with previously reported prevalence studies [4, 5, 48]. The clusters at these locations are likely caused by a complex interaction between the season [14], the local cattle holding density in these regions [13] and that most of the animal movements are over short to medium distances (i.e. <100 km) [49]. The simulated holding-level prevalence was also consistent with the 8.9% reported in a nationwide study of Swedish dairy herds [48]. The results are also in agreement with the individual-level prevalence reported in another previous nationwide study [5], although they are not directly comparable since the age categories are different. In that study, the observed prevalence was 3.5% in young stock (12–24 months) and 1.5% in adults (>36 months). In comparison, the simulated prevalence was 4.6–7.3% in young stock (3–12 months) and 1.2–2.1% in adults (>12 months). These findings suggest that the proposed transmission model captures important aspects of the VTEC O157 spatio-temporal dynamics on a national scale.

Incorporating both season, defined as the average temperature at the location of the holding, and spatial spread among neighbouring holdings, improved the agreement with observations compared to the model proposed by Widgren et al. [8] which lacked these characteristics. The results show that VTEC O157 transmission occurs at a much lower rate in northern Sweden, and that if infection was introduced, it became extinct over time. This is consistent with previous observations, as the VTEC O157 bacteria has rarely been detected in cattle originating from northern Sweden in prevalence studies [4, 5]. The environmental infectious pressure of VTEC O157 had the slowest decay during the summer, which leads to higher levels of the pathogen during that season. This finding is in agreement with the hypothesis that increased summertime VTEC O157 colonisation results from increased seasonal oral exposure to the pathogen [12].

Among the investigated control strategies, the one that most efficiently reduced the holding-level prevalence, was the combination of reducing both the indirect transmission rate and the rate of contribution to the environmental infectious pressure, per infected individual. These findings are consistent with previous modelling studies, where reducing the level at which infected cattle shed the pathogen [16, 50, 51], and decreasing the indirect transmission rate [52] has been shown to efficiently reduce the VTEC O157 prevalence in cattle. In the present study, a spatial coupling among proximal herds was included in the model to capture between-herd transmission of VTEC O157 unrelated to cattle movements, for example, via fomites such as vehicles or equipment, or by personnel. Although it is unrealistic to completely remove the spatial coupling among proximal herds, the results show a substantial reduction in prevalence after removing this transmission route, which highlights the importance of good external biosecurity to prevent spread and introduction of pathogens.

The results also show that the prevalence was, in principle, unaffected by network interventions, targeted on the measures in-degree, out-degree and ingoing contact chain as well as outgoing contact chain. This is in agreement with work by Zhang and Woolhouse [51] who reported that reducing movement related transmission has, at best, a modest impact in reducing the steady-state prevalence of VTEC O157. One approach to explore interventions involving animal trade is to generate a new contact network that incorporates the intervention of interest and then study the disease transmission in the new network [53]. However, a drawback of that approach is that it requires rules for how to rewire trading partners in the network; and those rules could disrupt other inherent properties of the livestock data that are important for the transmission process, for example, the population demographics. In this study, an alternative approach was used, which allowed the same network data to be used in all simulations. Instead of removing some movements from the network data to reduce between-herd spread, all movements were processed, but any infected cattle were replaced with susceptible cattle before adding them to the destination herd. Even this approach was not sufficient to efficiently reduce VTEC O157 within the Swedish cattle population. This also indicates a higher contribution to the between-herd spread from local spatial transmission compared to livestock movements.

The model was unable to completely capture the quarter-to-quarter fluctuations in the longitudinal observational study [6] used for calibration. This outcome might be explained by how the seasons were defined in this study, where the average temperature for the reference period 1961–1990 was used to classify seasons and not the actual temperature data for each year. This approach was used since reference data was readily available [14]. Although daily weather data was available, implementing a model informed by this detailed information at the herd-level would be computationally costly. Because precipitation and relative humidity might also affect conditions for bacterial replication and decay and have also been associated with increased VTEC O157 shedding [54], we suggest future research to explore the effect on the infection dynamics after incorporating high resolution spatio-temporal meteorological data in simulations. This is also supported by the global sensitivity analysis which suggests the seasonal parameters have a significant effect on the simulation behaviour. Another limitation of the disease spread model presented in this paper was that herd type was not included. It has been reported from several field studies that the production type e.g. dairy or beef, influences the risk for presence of VTEC O157 [6, 55, 56]. However, herd type was not available in the data that were used for modelling the population demographic and the temporal network.

The sensitivity analysis showed that the first order effect of the spatial coupling was not statistically significant for the simulation behaviour. A similar finding was reported by Zhang et al. [57] from fitting stochastic models for spread of VTEC O157 infection among Scottish cattle farms. However, in the present study, the sensitivity analysis showed that non-linear effects between the spatial coupling and other model parameters were statistically significant, suggesting that the spatial coupling should be included in the proposed VTEC O157 spread model. One limitation in the analysis of the spatial coupling is that exact coordinates were only available for 83.8% of the herds in the register data. This highlights the importance of a complete and validated cattle register to conduct data-driven disease spread simulations to evaluate potential risk factors and explore control strategies.

Using stochastic disease spread models is helpful to increase our understanding of the complex infection dynamics among interconnected herds. However, stochastic models introduce several challenges for inference with respect to model specification, parameter calibration, algorithm complexity and computational time. In this study, the parameter calibration was formulated as an optimisation problem using the Nelder–Mead algorithm to determine point estimates. However, since the model was stochastic, many realisations of the model had to be simulated for every iteration of the Nelder–Mead algorithm, and this increased the computational time. This suggests that parameterisation might require more sophisticated approaches e.g. to use a Bayesian methodology with suitable prior information on the parameter values. Another advantage of using Bayesian methods would be to estimate confidence intervals for the parameter values. The results presented in this study are based on fitting a data-driven pathogen-transmission model to a dataset consisting of repeated environmental sampling in 126 holdings, all located in southern Sweden. Due to the relatively limited size of that dataset, careful judgements must be considered when generalising to a national scale. However, the fact that the proposed methodology seems to capture the observed prevalence and infection dynamics of VTEC O157 on a national and regional scale, warrants further studies on other hazards of importance to animal and public health, e.g. *Salmonella* or antimicrobial resistance, using similar methodology.

It has been suggested that a substantial proportion of the VTEC O157 human cases could be prevented by vaccinating cattle against the pathogen [3]. The results of this study showed that reducing both the indirect transmission rate and the rate of contribution to the environmental infectious pressure, per infected individual, efficiently decreased the prevalence of VTEC O157 in the Swedish cattle population. This supports that vaccination could be a viable option to control VTEC O157 in cattle and hence have a public health benefit.
